# Development of a SAG1-based colloidal gold immunochromatographic strip for rapid serological detection of swine *Toxoplasma gondii*

**DOI:** 10.1186/s13071-026-07435-9

**Published:** 2026-05-21

**Authors:** Xin-xin Chen, Huiqin Sun, Yue Liang, Nannan Wang, Lu Fan, Liuhui Zhang, Guangxu Xing, Weitao Xie, Songlin Qiao

**Affiliations:** 1 Institute for Animal Health, Henan Academy of Agricultural Sciences, Key Laboratory of Animal Immunology of the Ministry of Agriculture, Zhengzhou, China; 2 College of Veterinary Medicine, Northwest A&F University, Yangling, China; 3 Shaanxi Shiyang Agricultural Technology Co., Ltd., Xi’an, China

**Keywords:** *Toxoplasma gondii*, Colloidal gold immunochromatographic strip, SAG1, Strip

## Abstract

**Background:**

Toxoplasmosis is a globally prevalent zoonosis caused by *Toxoplasma gondii* (*T. gondii*), a parasite that infects nearly all warm-blooded animals, including humans, cats, and pigs. Beyond incurring substantial economic losses to the swine industry, *T. gondii* infection poses a severe threat to public health, as pigs, which are key intermediate hosts for *T. gondii* transmission, serve as a major source of human infection via the food chain. Thus, developing specific, sensitive, rapid, and easy-to-perform detection methods for porcine *T. gondii* is of paramount importance for the prevention, control, and eventual eradication of this pathogen in swine populations.

**Methods:**

To address this need, a novel colloidal gold immunochromatographic strip (CGIS) was developed for the rapid serological detection of *T. gondii* infection in swine. The strip was constructed using recombinant surface antigen 1 (SAG1) expressed in a baculovirus-insect cell system, which ensures that the protein retains native antigenic epitopes. The purified SAG1 protein was conjugated to colloidal gold nanoparticles and loaded onto a conjugate pad, while staphylococcal protein A and a polyclonal anti-SAG1 antibody were immobilized on a nitrocellulose membrane to form the test line (T line) and control line (C line), respectively.

**Results:**

Clinical validation demonstrated that the developed CGIS exhibits high specificity, with no cross-reactivity to sera positive for 13 common porcine pathogens. The strip achieved a visual limit of detection of 1:25,600 dilution of positive serum. In parallel, a commercial enzyme-linked immunosorbent assay kit yielded positive results up to a 1:800 dilution according to the manufacturer’s diagnostic cutoff. Importantly, evaluation of 300 clinical porcine serum samples demonstrated a 98.3% concordance rate with the reference microscopic agglutination test.

**Conclusions:**

This SAG1-based CGIS provides a practical, user-friendly tool for large-scale surveillance and on-farm screening of porcine toxoplasmosis, thereby offering potential support for the prevention and control of *T. gondii* infection in pigs.

**Graphical abstract:**

**Figure:**
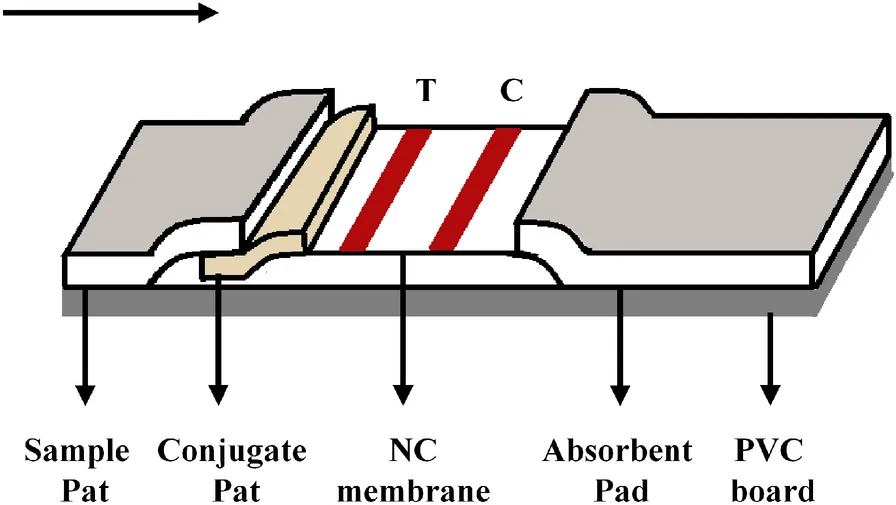

**Supplementary Information:**

The online version contains supplementary material available at https://doi.org/10.1186/s13071-026-07435-9.

## Background

Toxoplasmosis is a globally prevalent zoonosis caused by *Toxoplasma gondii* (*T. gondii*). As a zoonotic pathogen, *T. gondii* endangers swine health, impairs production efficiency, and imposes significant economic burdens on the pig industry worldwide [1]. Beyond its agricultural impact, *T. gondii* poses a severe public health threat. As crucial intermediate hosts in the *T. gondii* life cycle, pigs also serve as a major zoonotic reservoir, creating a serious public health risk through transmission routes such as the food chain. Chronic *T. gondii* infection is highly prevalent in swine populations worldwide, and its widespread occurrence has been well-documented. A systematic meta-analysis, which integrated seroprevalence data encompassing 148,092 pigs across 47 countries between 1990 and 2018, estimated a global seropositivity rate of approximately 19% [2], underscoring the endemic nature of this pathogen. In China, *T. gondii* was first isolated from cats and rabbits in Fujian Province in 1955 [3]. Since then, and particularly after 1979, a growing number of studies have been published, documenting its wide distribution across multiple regions, including the Northeast, North, Central, and East China [4–7]. Notably, effective prevention and treatment strategies for porcine toxoplasmosis remain limited. To date, no safe and effective commercial vaccine is available for the prevention of porcine toxoplasmosis. Clinically, *T. gondii* infections in pigs are often subclinical [8–10], and current intervention strategies mainly target acute cases, with sulfonamide drugs widely adopted as the standard therapeutic regimen [11]. However, sulfonamides have inherent limitations: while they can rapidly alleviate clinical symptoms, they fail to eliminate bradyzoites within tissue cysts. Delayed administration may even facilitate cyst formation in host tissues, leading to the infected pigs becoming asymptomatic carriers [12]. Furthermore, owing to concerns about drug residues, a withdrawal period of at least 1 month before slaughter is recommended for finishing pigs undergoing long-term sulfonamide treatment. Given the aforementioned limitations in vaccination and drug therapy, the serological monitoring of anti-*T. gondii* antibodies has emerged as a crucial indicator for detecting infection, providing essential tool to support the prevention and control of this disease.

*Toxoplasma gondii* proteins are categorized into four major classes: surface antigens (SAGs), rhoptry proteins (ROPs), microneme proteins (MICs), and dense granule proteins (GRAs). Among these, surface antigen 1 (SAG1), a well-characterized member of the SAGs, exhibits predominant and uniform distribution on the tachyzoite membrane and constitutes approximately 5% of the total protein content of *T. gondii* [13]. Functionally, SAG1 acts as the initial molecular mediator to interact with host cells during infection. Genetically, it is encoded by a single-copy gene that is highly conserved across different *T. gondii* strains [14, 15]. Notably, SAG1 exhibits the highest immunogenicity among all characterized tachyzoite proteins. It can elicit specific antibody responses. These responses account for up to 50% of the total humoral immune responses induced by *T. gondii* infection [10]. These attributes render SAG1 a promising candidate for the development of both diagnostic antigens and vaccines. In fact, SAG1 has been widely employed in multiple serological assays, such as enzyme-linked immunosorbent assay (ELISA) and immunofluorescence assays (IFA), for detecting *T. gondii*-specific antibodies [16–21]. However, these conventional methods often require specialized equipment and trained personnel, limiting their utility in field settings and large-scale screening. Therefore, developing rapid, equipment-free diagnostic tools based on SAG1 remains an ongoing need.

In this study, we developed a colloidal gold immunochromatographic strip (CGIS) targeting the SAG1 antigen for serodiagnosis and epidemiological surveillance of *T. gondii* infections. Compared with commercially available kits, the developed strip demonstrates favorable analytical sensitivity and operational simplicity, making it a practical tool for large-scale field-based serological testing in swine production systems.

## Methods

### Cells and sera

Sf9 and High Five™ insect cell lines were obtained from the China Center for Type Culture Collection (CCTCC). Porcine serum samples negative and positive for *T. gondii* antibodies were kindly provided by Prof. Yurong Yang (Henan Agricultural University). The following sera were maintained in our laboratory: 64 negative serum samples from specific-pathogen-free (SPF) swine farms, 300 clinical serum samples from diseased pigs, and positive control sera for antibodies against *Cryptosporidium* spp., *Trichinella spiralis*, *Isospora suis*, porcine reproductive and respiratory syndrome virus (PRRSV), classical swine fever virus (CSFV), pseudorabies virus (PRV), porcine epidemic diarrhea virus (PEDV), and porcine parvovirus (PPV), *Streptococcus suis*, *Escherichia coli*, and *Mycoplasma hyopneumoniae*. All serum samples were stored at −80 °C upon collection to avoid repeated freeze–thaw cycles.

### Expression and purification of *Toxoplasma gondii* SAG1 protein

The SAG1 genes of *T. gondii* (GenBank: X14080.1) were synthesized and cloned into the pFastBac plasmid by GenScript Biotech Corporation (Suzhou, China). A recombinant plasmid pFastBac-SAG1 was subsequently constructed and transfected into DH10Bac cells. Recombinant bacmids were selected through blue–white screening, and the recombinant bacmid DNA was extracted. The bacmids were subsequently transfected into Sf21 insect cells using a lipid-based transfection reagent. The first-generation viral supernatant was harvested, followed by three consecutive rounds of viral propagation for amplification to generate a high-titer fourth-generation viral stock. Protein expression was verified by sodium dodecyl sulfate–polyacrylamide gel electrophoresis (SDS–PAGE) and western blot analysis. For SDS–PAGE, protein samples were separated on 12% polyacrylamide gels and visualized by Coomassie Brilliant Blue staining. For western blot, the separated proteins were transferred onto polyvinylidene fluoride (PVDF) membranes. After blocking with 5% skim milk in phosphate-buffered saline with Tween 20 (PBST), the membranes were probed with either His-horseradish peroxidase (HRP)-conjugated anti-His antibody (1:5,000) or *T. gondii*-positive swine serum (1:100). In the case of swine serum incubation, HRP-conjugated goat anti-swine IgG (1:5,000) was employed as the secondary antibody. Protein bands were visualized using an enhanced chemiluminescence (ECL) substrate.

For recombinant SAG1 protein purification, the fourth-generation viral supernatants were used to infect Sf21 cells. The culture supernatant was collected by centrifugation at 3,000× *g* for 10 min and filtered through a 0.45-μm membrane. The recombinant SAG1 protein was purified using a two-step chromatographic approach: affinity chromatography with a His-tag purification system, followed by gel filtration chromatography to enhance purity. For affinity chromatography, the protein was eluted with a buffer containing 300 mM imidazole. The eluted fraction was then concentrated and loaded onto the gel filtration column pre-equilibrated with phosphate-buffered saline (PBS). The eluted protein was confirmed by SDS–PAGE and western blot. The protein concentration was determined using the bicinchoninic acid (BCA) assay. The purified protein was aliquoted and stored at −80 °C for subsequent polyclonal antibody production and the preparation of colloidal gold conjugates for downstream applications.

To assess the antigenic reactivity of the purified SAG1 protein, a dot blot assay was performed. Briefly, 2 μL of the purified recombinant SAG1 protein (1 mg/mL) was spotted onto a nitrocellulose membrane and air-dried. The membrane was blocked with 5% skim milk in PBST at 37 °C for 1 h, then incubated with *T. gondii*-positive swine serum (1:100) for 1 h at room temperature. After washing, the membrane was incubated with HRP-conjugated goat anti-swine IgG (1:5,000) for 45 min, and signals were visualized using ECL chemiluminescence substrate.

### Preparation and purification of polyclonal antibodies

To generate polyclonal antibodies [22], the previously expressed and purified SAG1 protein (in monomeric form) was utilized as the immunogen to immunize 6-week-old healthy New Zealand White rabbits via subcutaneous multipoint injection with 100 μg per rabbit. The rabbits were immunized a total of four times at 2-week intervals. Then, blood was collected to determine the antibody titers by ELISA. When antibody titers against SAG1 exceeded 1:1 × 10^7^, whole blood was collected from the rabbits. The blood was allowed to clot at 37 °C for 2 h, followed by overnight incubation at 4 °C. The serum containing polyclonal antibodies (pAbs) against SAG1 was then isolated by centrifugation at 3,000*g* for 10 min.

For antibody purification, a sodium sulfate precipitation method was employed [23]. Briefly, 1 mL of antiserum was slowly added dropwise to a 18% (w/v) anhydrous sodium sulfate solution with continuous stirring. Then the mixture was incubated at 37 °C for 2 h. After incubation, the mixture was centrifuged at 10,000× *g* for 15 min, after which the supernatant was discarded. The precipitated antibodies were resuspended in 1 mL of ultrapure water and dialyzed against 0.9% physiological saline (NaCl solution) three times to remove residual salts. The purified polyclonal antibodies against SAG1 were subsequently used for colloidal gold conjugation.

The endpoint titers of serum antibody were determined by an indirect ELISA. Briefly, 96-well microplates were coated with purified SAG1 protein and incubated overnight at 4 °C. After blocking with 5% skim milk, serial twofold dilutions of rabbit antiserum obtained after the fourth immunization were added and incubated for 1 h. Following washing, the plates were incubated with His-horseradish peroxidase (HRP)-conjugated goat anti-rabbit IgG secondary antibody. After color development and termination, the absorbance was read at 450 nm.

### Synthesis and characterization of colloidal gold nanoparticles

Colloidal gold nanoparticles were synthesized via the trisodium citrate reduction method [22]. Briefly, 1 mL of 1% (w/v) chloroauric acid (HAuCl_4_) was added to 100 mL of deionized water under vigorous stirring and boiling. Subsequently, 1.6 mL of a 10 g/L trisodium citrate solution was rapidly injected into the boiling mixture, which was continuously heated until the solution stabilized to a bright red color, an indicator of the formation of well-dispersed colloidal gold nanoparticles. Following synthesis, the colloidal gold suspension was cooled to room temperature, filtered through a 0.22-μm membrane, and stored at 4 °C in the dark.

For the characterization of the colloidal gold nanoparticles, their optical properties were analyzed using ultraviolett–visible (UV–Vis) spectroscopy to acquire an absorbance spectrum. UV–Vis measurements were performed using an Omega optical spectroscopy system (Omega Optical, Inc., Brattleboro, VT, USA) over a wavelength range of 400–700 nm, and the maximum absorbance wavelength (*λ*max) was recorded to confirm nanoparticle formation. The particle size distribution was determined using dynamic light scattering (DLS) analysis with a Malvern Zetasizer Nano ZS90 instrument (Malvern Panalytical Ltd., Malvern, UK).

### Optimization of pH and labeling parameters

To determine the optimal pH for protein-colloidal gold conjugation, the colloidal gold suspension was adjusted to a series of pH values (ranging from 5.0 to 9.0) using 0.2 M K_2_CO_3_. The colloidal gold suspension that exhibited no visible aggregation (retained bright red color) and showed maximal absorbance at 530 nm was used as the basis for selecting the optimal pH, and its corresponding pH was selected as the optimal one.

To determine the optimal labeling concentration of the SAG1 protein, a salt stability assay was conducted. Serial twofold dilutions of SAG1 protein with 10-μL initial volume in ultrapure water were prepared in a 96-well plate. Then, 100 μL of pH-adjusted colloidal gold was added to each well, and the plate was incubated at room temperature for 5 min. Subsequently, 100 μL of 10% NaCl (w/v) was added to each well. The plate was gently shaken and incubated for 10 min. The stability of the colloidal gold in each well was assessed by visual observation for color change (a shift from red to blue/gray indicated aggregation) and absorbance measurement at 530 nm using a microplate reader. The lowest protein concentration that maintained a bright red color and yielded the maximum absorbance value was defined as the optimal labeling concentration.

### Assembly of CGIS and result interpretation

**Fig. 1:**
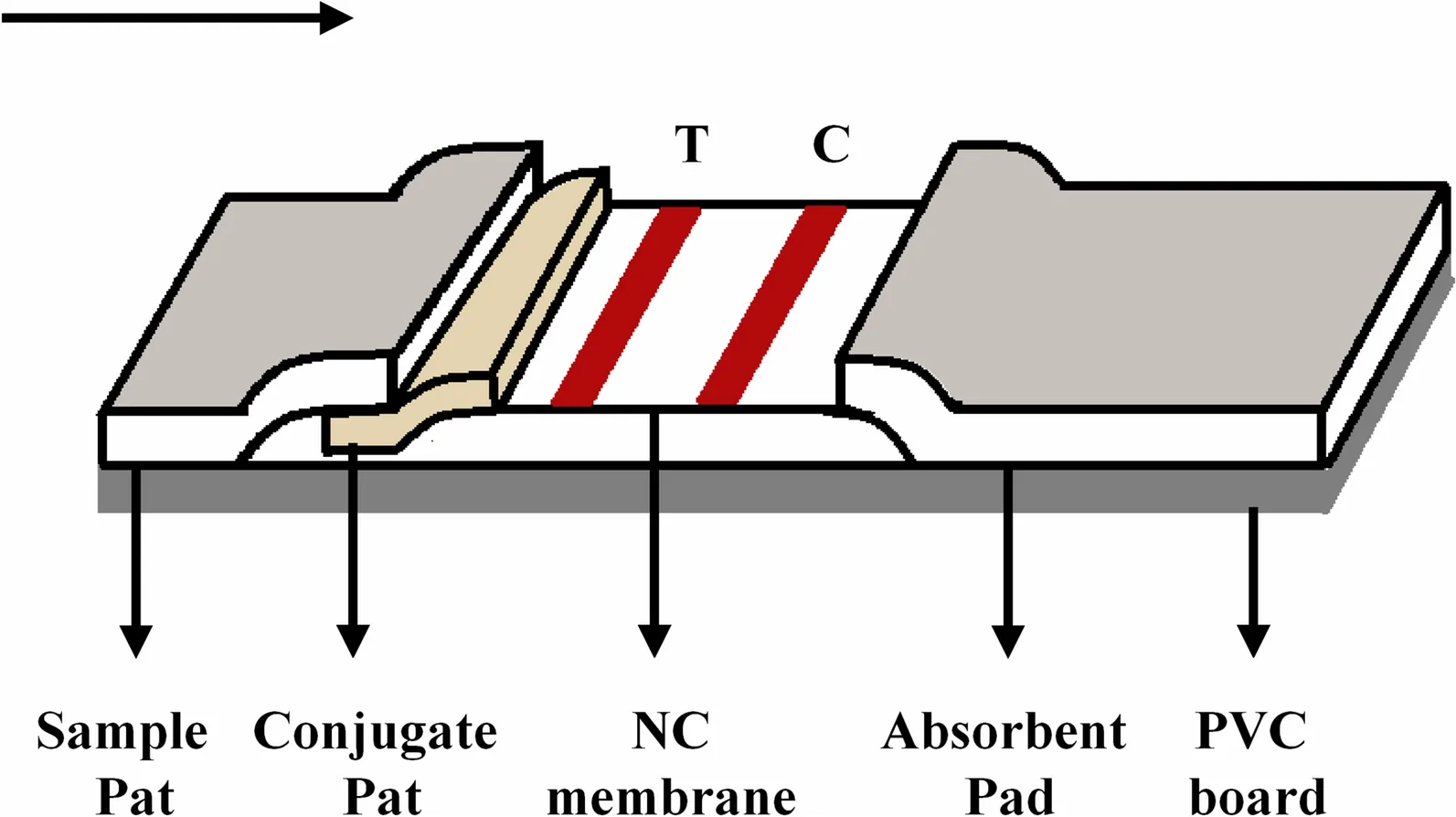
Schematic illustration of the CGIS assembly. Components: sample pad; conjugate pad (precoated with gold-labeled anti-SAG1); NC membrane, where T and C denote the T line (SAG1 monomer) and the control (C) line (anti-species IgG), respectively; and absorbent pad

As illustrated in Fig. 1, CGIS were assembled by sequentially laminating the following components onto a PVC backing card with a 2 mm overlap between adjacent components: a sample pad, a conjugate pad (precoated with gold-labeled SAG1), a nitrocellulose (NC) membrane (pre-immobilized with staphylococcal protein A (SPA) at the test line (T line) and goat anti-rabbit IgG at the control lines (C line)), and an absorbent pad. The assembled card was cut into 2.8-mm-wide strips using a CM4000 guillotine cutter. These strips were housed in plastic cassettes and then stored in aluminum foil bags with desiccant at room temperature.

For testing, 100 μL of the serum sample diluted 1:200 in PBS was applied to the sample well. The results were interpreted after a 10-min incubation at room temperature. A valid test was confirmed by the appearance of a distinct red band at the C line, indicating proper fluid flow and reagent functionality. A sample was considered positive if two red bands were visible at both the C and T lines, whereas a negative result was defined by the presence of a single band only at the C line. Furthermore, a semiquantitative correlation was observed, as the color intensity of the T line was proportional to the specific antibody titer within the assay’s linear detection range, with darker T lines corresponding to higher antibody concentrations.

### Evaluation of sensitivity, specificity, and diagnostic concordance

To determine the specificity of the colloidal gold strip, it was evaluated using porcine serum samples confirmed positive for antibodies against 13 common porcine pathogens, including *Cryptosporidium* spp., *Trichinella spiralis*, *Isospora suis*, PRRSV, CSFV, PRV, PEDV, PCV2, FMDV, PPV, *Streptococcus suis*, *Escherichia coli*, and *Mycoplasma hyopneumoniae*. Additionally, 64 clinical serum samples from SPF swine farms were included as negative controls.

To determine sensitivity, *T. gondii*-positive sera were serially diluted in twofold increments, and the limit of detection (LOD) of the colloidal gold strip was compared with that of a commercial ELISA kit.

For diagnostic concordance analysis, 300 porcine clinical serum samples were subjected to parallel testing using the colloidal gold strip and the microscopic agglutination test (MAT). The concordance rate between the two methods was calculated to evaluate consistency in detection outcomes. MAT was performed according to the standard protocol described previously [24–26], with minor modifications. Briefly, *T. gondii* tachyzoites (RH strain) were harvested, and fixed with formalin as the detection antigen. Serum samples were diluted 1:25 with PBS containing 0.2 M 2-mercaptoethanol (2-ME) in V-bottomed microplates. Subsequently, an equal volume of the antigen suspension was added to each well. After thorough mixing, the plates were incubated overnight at 37 °C. The plates were then tilted to observe the agglutination pattern. A positive result was characterized by a diffuse agglutination layer covering the well bottom, while a negative result was indicated by the formation of a compact, clear-edged button of tachyzoites.

### Stability assessment

The stability and repeatability of the colloidal gold strip were systematically evaluated. To assess repeatability, three independent batches of strips produced using different protein lots were tested against fourfold serially diluted *T. gondii*-positive standard sera and a negative serum, with all samples run in triplicate. For stability analysis, strips stored in sealed aluminum foil pouches at room temperature were evaluated over periods of 2, 4, 6, 8, and 12 months using the same serum panel. The T line was scanned with a TSR3000 membrane strip reader (Shenzhen Shenfen Instrument Manufacturing Co., Ltd., Shenzhen, China) to obtain relative optical density (ROD) values. The cutoff value was set at 0.2, calculated as the mean T/C ratio of 50 negative serum samples plus three standard deviations (mean + 3SD). The coefficient of variation (CV) for each dilution was calculated as the standard deviation divided by the mean ROD value from three independent tests. A CV value below 15% was considered indicative of acceptable repeatability, while stability was defined as the maintenance of baseline detection performance throughout the storage period.

## Results

### Expression and purification of *T. gondii* SAG1 protein

**Fig. 2:**
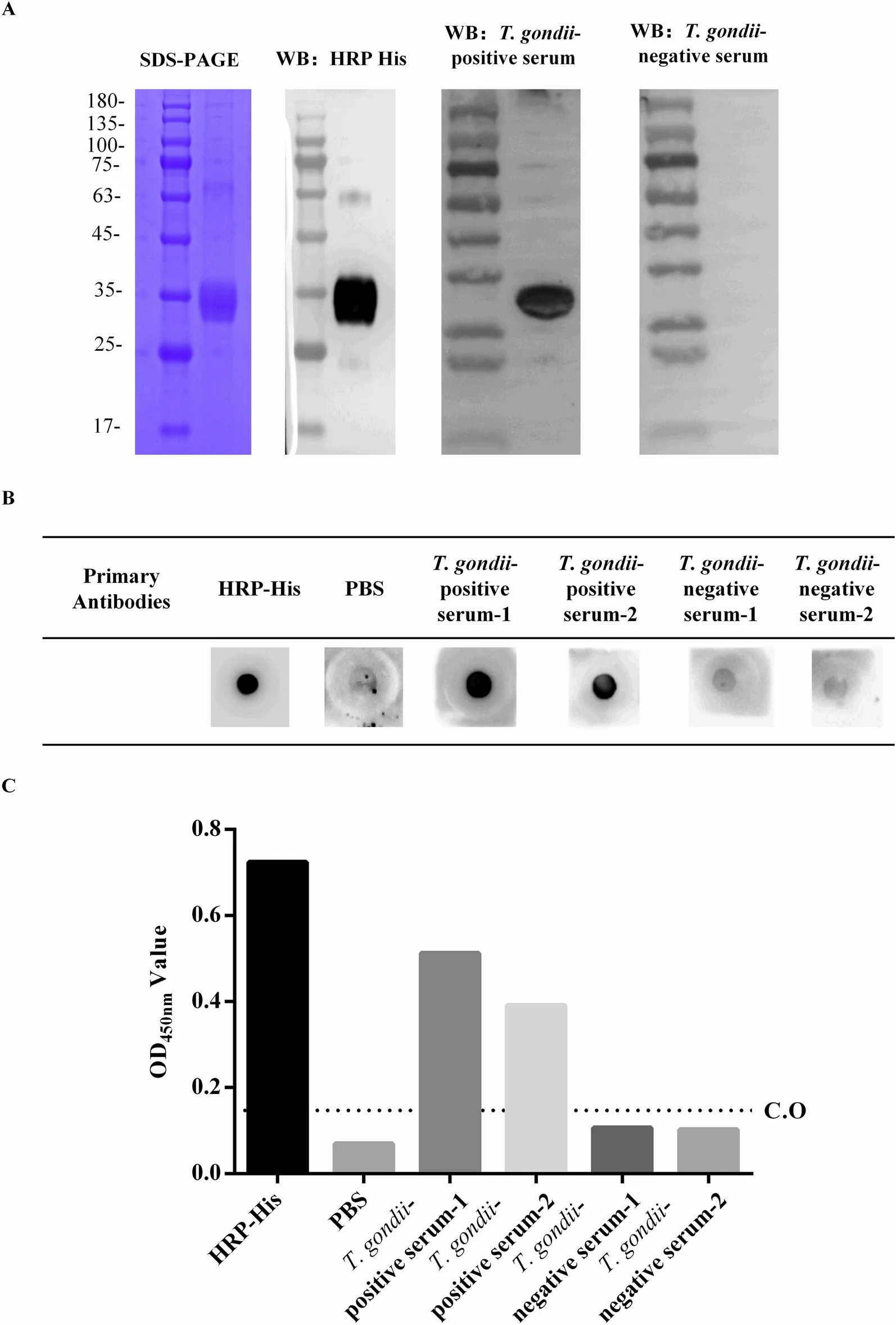
Expression, purification, and reactivity characterization of recombinant SAG1 protein. **A** The purified SAG1 was verified by SDS–PAGE and western blot analysis. Western blot was probed with anti-His-HRP, *T. gondii*-positive serum, and *T. gondii*-negative serum, respectively. **B** Dot blot analysis of purified SAG1 protein reactivity. **C** ELISA assessment of SAG1 immunoreactivity. Microplate wells were coated with SAG1 protein (200 ng/well) and incubated with PBS, anti-His-HRP, *T. gondii*-positive serum, or *T. gondii*-negative serum, followed by measurement of the absorbance at 450 nm (OD_450_)

To obtain the SAG1 protein in a near-native conformation, the baculovirus-insect cell expression system was utilized [27, 28]. Western blot analysis confirmed that a recombinant 35-kDa protein, consistent with the expected molecular weight of SAG1 [15], was successfully expressed in the third-passage viral supernatant (Fig. 2A). For large-scale production of SAG1, the fourth-passage viral stock was used to infect Sf21 insect cells. The recombinant SAG1 protein was subsequently purified using nickel affinity chromatography under native conditions, yielding a highly purified product with a protein concentration of 3.5 mg/mL as quantified via BCA assay. The antigenic reactivity of the purified protein was further validated by dot blot assay (Fig. 2B) and ELISA (Fig. 2C). The dot blot assay showed clear positive signals when the purified SAG1 was probed with HRP-conjugated anti-His antibody or *T. gondii*-positive serum, while no signal was observed with PBS or *T. gondii*-negative serum. In the ELISA, microplate wells coated with the purified SAG1 protein exhibited significantly higher absorbance values at 450 nm (OD_450_) compared with controls. These results collectively demonstrated that the purified SAG1 retained specific binding activity to anti-SAG1 polyclonal antibodies.

### Preparation of polyclonal antibodies

**Fig. 3:**
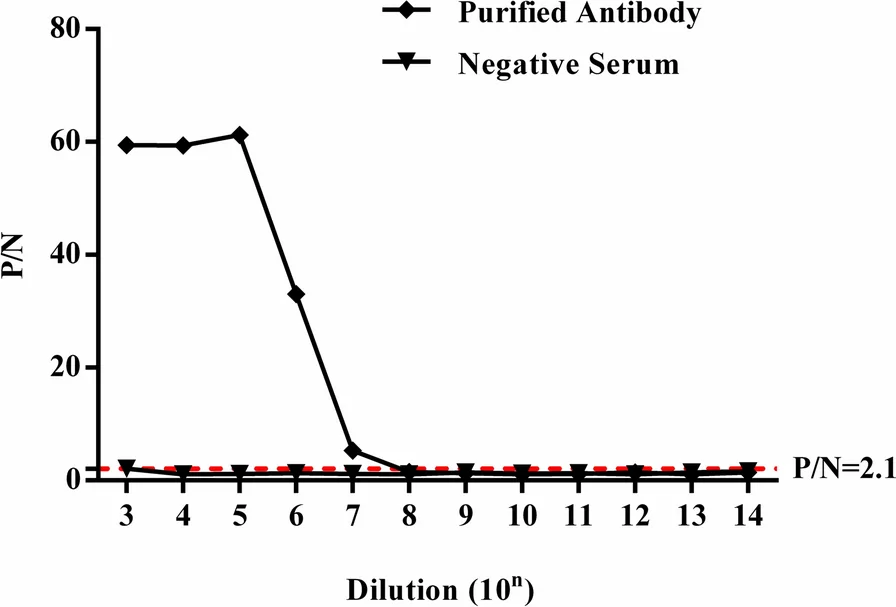
Determination of polyclonal antibody titers. Endpoint titers (expressed as log_10_) in rabbits after the fourth immunization were measured by indirect ELISA. The dashed line indicates the positive/negative (P/N) cutoff value

PAbs against the recombinant purified *T. gondii* SAG1 were generated by immunization of 6-week-old SPF New Zealand White rabbits. After the primary immunization, booster immunizations were administered at 2-week intervals to further enhance the immune response. Subsequently, serum antibody titers were quantified using indirect ELISA (Fig. 3). After the fourth immunization, a final serum antibody titer of 1 × 10^7^ was achieved. This outcome further confirms the immunogenicity of the *T. gondii* SAG1 antigen used in this study. The anti-SAG1 pAbs were further purified using sodium sulfate precipitation for subsequent experiments.

### Preparation of gold-labeled protein conjugates

**Fig. 4:**
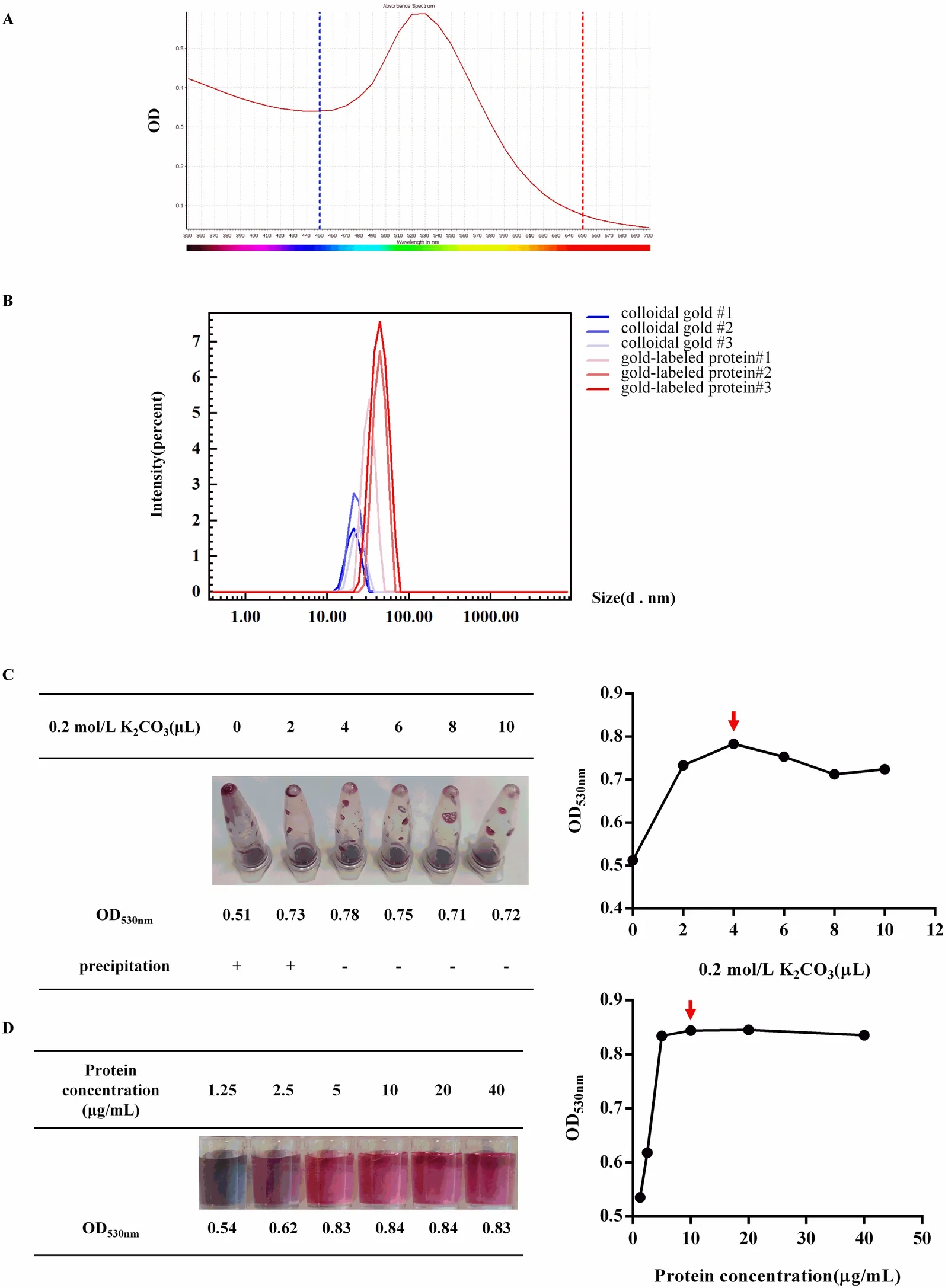
Optimization of colloidal gold conjugation parameters. **A** UV–Vis absorbance spectrum of synthesized colloidal gold nanoparticles exhibiting a peak at 530 nm. **B** Hydrodynamic diameter distribution of colloidal gold and SAG1-gold conjugates determined by DLS. **C** The UV–Vis absorbance spectrum of the colloidal gold nanoparticles was determined after optimizing the pH by adding indicated volume of K_2_CO_3_. The arrow indicates the concentration of K_2_CO_3_ at the optimal pH. **D** Minimal stabilizing SAG1 concentration was determined by salt aggregation assay. The UV–Vis absorbance spectrum of the colloidal gold nanoparticles was determined after incubation with serial dilutions of SAG1 protein. The arrow indicates the minimal concentration that prevents aggregation

Colloidal gold nanoparticles were synthesized and characterized prior to conjugation [22]. UV–Vis spectrophotometry exhibited a maximum absorbance at 530 nm (Fig. 4A). Dynamic light scattering (DLS) using a Malvern Zetasizer Nano ZS90 further revealed a mean particle size of 22 nm, consistent with the size range required for stable immunochromatographic reactions (Fig. 4B). To optimize colloidal stability [29], the pH of the gold suspension was adjusted using 0.2 mol/L (M) K_2_CO_3_, with six pH gradients prepared by adding 0, 2, 4, 6, 8, or 10 μL of K_2_CO_3_ to 1 mL of colloidal gold, respectively. Stability was assessed by monitoring aggregation (visible precipitation) and measuring optical density at 530 nm (OD_530 nm_). The addition of 4 μL of 0.2 M K_2_CO_3_ resulted in the highest OD_530 nm_ and no visible precipitation (Fig. 4C). Thus, the pH value of the colloidal gold nanoparticle at this concentration was identified as the optimal pH for subsequent protein conjugation.

The minimum stabilizing concentration of the SAG1 protein was determined using a salt stability assay. Serial dilutions of SAG1 protein (0–20 μg/mL) were incubated with pH-adjusted colloidal gold (Fig. 4D). At a SAG1 concentration of 5 μg/mL, the colloidal gold retained its characteristic red color and stable dispersion. No significant changes in OD_530 nm_ or aggregation were observed even with further increases in protein concentration, indicating this as the minimum stabilizing concentration. DLS analysis was conducted to validate successful conjugation, revealing an increased mean particle size of 44 nm for the gold-protein conjugate. On the basis of these results, 5 μg/mL was finalized as the optimal SAG1 concentration for colloidal gold labeling.

### Sensitivity and specificity evaluation of the strip

The analytical sensitivity of the CGIS was evaluated using serially diluted positive serum samples. The colloidal gold strip exhibited a clear visual LOD up to a dilution of 1:25,600 (Additional file 1: Supplementary Fig. S1A). In parallel, the same dilution series was analyzed using a commercial ID-vet *T. gondii* ELISA kit, which yielded positive results up to a dilution of 1:800 according to the manufacturer's diagnostic cutoff (Additional file 1: Supplementary Fig. S1B). These results demonstrate that the developed CGIS possesses favorable analytical sensitivity, which is more than sufficient for clinical and field-based applications.

For the specificity evaluation, 64 serum samples obtained from SPF swine farms, which were confirmed to be *T. gondii*-negative, were tested to assess false-positive rates. No false-positive results were observed for either the developed strip or the commercial ID-vet *T. gondii* ELISA kit (Additional file 2: Supplementary Fig. S2A, B), demonstrating 100% specificity within this limited sample set.

To further evaluate the specificity, cross-reactivity was tested with sera positive for antibodies against common porcine pathogens, which were categorized into four groups: parasites, including *Cryptosporidium* spp., *Trichinella spiralis*, and *Cysticercus cellulosae*; viruses, including PRRSV, CSFV, PRV, PEDV, and PPV; bacteria, including *Streptococcus suis* and *Escherichia coli*; and a mycoplasma species, *Mycoplasma hyopneumoniae*. As shown in Additional file 3: Supplementary Fig. S3, the colloidal gold strip reacted exclusively with *T. gondii*-positive sera, with no cross-reactivity observed against serum samples containing antibodies specific to the other tested pathogens.

To assess the diagnostic concordance between the CGIS and the MAT, 300 clinical porcine serum samples were tested in parallel with both methods. The MAT identified 267 samples as negative, while the colloidal gold strip detected 262 negative samples. The CGIS showed a concordance rate of 98.3% (Table 1). The five samples that tested negative by MAT but positive by the strip were further analyzed by western blot, all of which showed positive reactivity with SAG1 (data not shown), suggesting that the CGIS may detect low-titer antibodies that are below the MAT detection threshold.

**Table 1:** Concordance analysis between CGIS and MAT

MAT	Strip		
	Positive sera	Negative sera	Total
Positive sera	33	0	33
Negative sera	5	262	267
Total	38	262	300
Concordance rate	98.3%		

### Reproducibility and stability evaluation of the strip

To assess the reproducibility and stability of the developed strip, the batch-to-batch consistency and the long-term storage stability of the strips were systematically evaluated.

For reproducibility assessment, three independently manufactured batches of strips were used to test against five *T. gondii*-negative and five *T. gondii*-positive serum samples, with all tests performed in triplicate. The intrabatch and interbatch coefficients of CV were calculated on the basis of the consistency of detection results (Table 2). Both intrabatch CV and interbatch CV were 0%, indicating that the developed strip exhibited good reproducibility and consistent performance across production batches under the tested conditions.

**Table 2:** Reproducibility assessment across different production batches

Test strip batches	Number 1			Number 2			Number 3		
Replicates	1	2	3	1	2	3	1	2	3
Positive sera	5	5	5	5	5	5	5	5	5
Negative sera	5	5	5	5	5	5	5	5	5
Intrabatch CV (%)	0%			0%			0%		
Interbatch CV (%)	0%								

For the stability assessment, strips from a single production batch were sealed in desiccant-containing aluminum foil bags and stored at room temperature. At 2-month intervals (2, 4, 6, 8, and 12 months after storage), subsets of strips were tested with serially diluted *T. gondii*-positive sera (Table 3). Even after 12 months of storage, the strips retained the LOD of 1:25,600 with no observable decrease in sensitivity or specificity. These results demonstrate that the developed strip maintain its initial detection performance for at least 12 months under the tested ambient storage conditions.

**Table 3:** Stability performance under ambient temperature storage

Storage time (months)	2	4	6	8	12	CV (%)
Dilution factor	T line/C line signal ratio (%)					
1:100	74.7	76.0	75.5	74.0	72.4	1.69
1:400	63.4	67.2	64.4	61.7	63.7	2.80
1:1,600	22.3	25.1	24.7	22.9	21.8	5.61
1:6,400	12.7	14.3	15.7	14.3	14.7	6.74
1:25,600	6.8	7.4	7.1	7.3	6.5	4.72
*N*	0	0	0	0	0	0

Note: N, negative serum control.

## Discussion

*Toxoplasma gondii* was identified as a priority zoonotic pathogen by the World Health Organization owing to its significant impact on public health, particularly in pregnant women, fetuses, and immunocompromised individuals[30–33]. Pigs are recognized as one of the most important intermediate hosts for *T. gondii*, and consumption of undercooked or raw pork is a major route of human infection. Moreover, *T. gondii* imposes significant economic burdens on the swine industry. The lack of effective vaccines, coupled with the limitations of conventional therapeutic regimens, underscores the critical role of accurate and accessible diagnostic approaches in effective disease control. However, established serological methods such as MAT and ELISA are often constrained in field settings by operational complexity, high cost, and stringent technical expertise requirements [34–36]. This gap highlights an urgent need for diagnostic tools that are not only accurate but also suitable for on-farm, point-of-care testing. CGIS has emerged as a promising solution to address this unmet need, offering inherent advantages of rapid result acquisition, operational simplicity, and minimal instrumental dependency [37, 38]. In this study, we successfully developed a novel CGIS based on the recombinant SAG1 of *T. gondii*, which exhibits high sensitivity, specificity, reproducibility, and stability. These characteristics make it a promising tool for large-scale epidemiological surveillance and on-farm screening of porcine toxoplasmosis.

The selection of target antigens is a critical determinant of the diagnostic performance of CGIS. SAG1 is an immunodominant protein abundantly expressed on the tachyzoite membrane, responsible for initiating host cell attachment [10]. Its high immunogenicity and ability to elicit a robust humoral immune response, accounting for a significant proportion of host antibody production, render it an ideal target for serodiagnosis [10, 39]. Thus, SAG1 has been widely explored in various immunoassays in previous studies, such as ELISA, IFA, and other serological assays [18, 20, 40–44]. Our study further confirms the validity of SAG1 as a diagnostic target by successfully applying it to the CGIS platform, suggesting its potential for broader diagnostic applications.

The choice of an appropriate expression system is a critical determinant of the diagnostic efficacy in serological assays. In this study, we employed a baculovirus-insect cell expression system to ensure the production of SAG1 protein with authentic antigenic epitopes. Although *E. coli* expression is more cost-effective, *T. gondii* SAG1 is an immunodominant surface antigen whose reactivity depends heavily on proper disulfide bond formation and conformational epitopes [45, 46]. Recombinant SAG1 expressed in *E. coli* frequently forms insoluble inclusion bodies and lacks critical posttranslational modifications, often leading to suboptimal diagnostic performance. In contrast, the baculovirus-insect cell system provides a eukaryotic folding environment that enables proper protein maturation and preserves native antigenic epitopes. This structural fidelity offers a tangible diagnostic advantage, particularly in accurately detecting natural antibodies in clinical sera [47, 48]. This approach proved advantageous, enabling the generation of high-affinity polyclonal antibodies (final titer of 1:102,400) through a multidose immunization protocol. In addition, the prepared SAG1 was purposefully applied in the development of the test strip: the purified SAG1 monomer served as the capture antigen at the T line, and the anti-SAG1 polyclonal antibody was used at the C line. This design was validated to be effective in ensuring the strip’s diagnostic performance in our experiments.

Our evaluation demonstrates that the optimized SAG1-based CGIS exhibits favorable performance. In terms of sensitivity, the CGIS achieved a visual LOD of 1:25,600, demonstrating a capability for detecting low-titer antibodies, which may be useful for identifying subclinical or chronic carriers. The developed strip also displayed favorable analytical specificity, showing no cross-reactivity with sera positive for other common swine pathogens. Furthermore, validation against MAT yielded a 98.3% concordance rate. Although minor discrepancies were observed (5/300), western blot confirmation of these samples as true positives further underscores the high diagnostic reliability of our strip. The test strip also exhibits considerable stability: it maintains its initial detection sensitivity for 12 months at room temperature with minimal batch-to-batch variation (CV < 10%). Taken together, these results support its suitability for use in routine field-based screening under ambient storage conditions.

However, several limitations of this study should be acknowledged. First, the CGIS provides semiquantitative results based on visual interpretation, and precise antibody titer determination remains the domain of quantitative methods such as ELISA. Second, the performance of the strip has not been verified using a large number of *T. gondii* isolates, which may affect its broad applicability in regions with genetically diverse parasite populations. Further studies using larger and geographically diverse sample sets are warranted to fully validate its clinical utility. Third, stability was assessed under controlled room temperature storage with desiccant; performance under high-temperature or high-humidity field conditions was not tested.

Despite these limitations, the developed strip represents a practical and reliable tool for rapid on-farm screening and large-scale serosurveillance.

## Conclusions

We have developed a novel recombinant SAG1-based CGIS for the serological detection of *T. gondii* in swine. The assay combines the operational ease of a strip test with performance metrics that meet the requirements for field applications and outperform conventional laboratory methods in certain aspects. Its observed sensitivity, specificity, and stability make it a practical and powerful tool for large-scale sera-surveillance and on-farm screening, thereby contributing to more effective control and prevention strategies for porcine toxoplasmosis.

## Supplementary Information

**Additional file 1**: **Figure S1**. Sensitivity evaluation of the developed strip. (**A**) The analytical sensitivity of the developed strip was determined using two-fold serially diluted *T. gondii*-positive serum from 1:200 to 1:204,800. Results were visually interpreted at 10 min post-incubation. (**B**) Parallel sensitivity testing with the commercial *T. gondii* ELISA kit (ID-vet, France) was performed. Asterisks (★) indicate the LOD for each method.

**Additional file 2**: **Figure S2**. Specificity validation of the developed strip. (**A**) Specificity testing was performed using 64 serum samples from SPF swine farms. (**B**) Specificity validation was conducted on the same 64 SPF swine serum samples using the commercial ID-vet *T. gondii* ELISA kit.

**Additional file 3**: **Figure S3**. Cross-reactivity assessment of the developed strip. The cross-reactivity of strip was tested using swine sera confirmed positive for antibodies against the common porcine pathogens as indicated: *Cryptosporidium* spp., *T. spiralis*, *C. cellulosae*, PRRSV, CSFV, PRV, PEDV, PCV2, FMDV, PPV, *S. suis*, *E. coli*, and *M. hyopneumoniae*.

## Data Availability

The datasets supporting the conclusions of this article are available within the article.

## Abbreviations

*T. gondii*: *Toxoplasma gondii*
SAGs: Surface antigens
CGIS: Colloidal gold immunochromatographic strip
ELISA: Enzyme-linked immunosorbent assay
MAT: Microscopic agglutination test
IFA: Immunofluorescence assays
SDS–PAGE: Sodium dodecyl sulfate–polyacrylamide gel electrophoresis
pAbs: Polyclonal antibodies
HRP: His-horseradish peroxidase
SPA: Staphylococcal protein A
T line: Test line
C line: Control lines
LOD: Limit of detection
CV: Variation
